# The special extract ERr 731^®^ from the root of rhapontic rhubarb (*Rheum rhaponticum*): efficacy in headache/migraine and further climacteric complaints in women in the perimenopause

**DOI:** 10.3389/fphar.2026.1831343

**Published:** 2026-06-23

**Authors:** Peter W. Heger, Ilka Meinert, Dirk Hotz

**Affiliations:** Health Research Services GmbH, Ubstadt-Weiher, Germany

**Keywords:** ERr 731^®^, menopause, non-hormonal treatment, rhapontic rhubarb extract, *Rheum rhaponticum*, vasomotor symptoms

## Abstract

**Background:**

Menopause is associated with neuroendocrine changes and a variety of climacteric symptoms. While hormone replacement therapy (HRT) remains a standard treatment, safety concerns increase global interest in non-hormonal alternatives like ERr 731®, an extract from *Rheum rhaponticum* to alleviate climacteric complaints.

**Objective:**

To evaluate the long-term effectiveness of ERr 731® in reducing menopausal climacteric symptoms during a 12-week randomized controlled (RCT) trial followed by a 52-week open-label observational study (OS).

**Methods:**

One hundred and twelve perimenopausal women (aged 45–55 years) with climacteric complaints (Menopausal Rating Scale (MRS) ≥18) participated in this RCT while eighty-nine of these study participants continued in the OS. During the RCT patients received either ERr 731® (4 mg daily) or a placebo. During the OS, all participants received ERr 731® and were assessed every 13 weeks for headache/migraine, dizziness, paresthesia, fluor vaginalis, and general well-being. Descriptive statistics and exploratory t-tests compared symptom severity between day 0 and day 84 as well as day 364.

**Results:**

During the RCT, climacteric complaints were significantly reduced in the ERr 731® group compared to placebo. Symptom severity also decreased across all domains from baseline to week 52 of the OS. Headache/migraine and paresthesia improvements were notable. Since all participants received ERr 731® during the OS, no significant differences between the prior ERr 731® and placebo groups were present (*p* > 0.05). Dizziness reduction remained significant between groups (*p* = 0.0086). General well-being improved markedly, with >90% of participants reporting to be “good” or “very good spirits” at week 52.

**Conclusion:**

ERr 731® demonstrated sustained symptom relief and improved well-being over 52 weeks, supporting its role as a non-hormonal option for managing climacteric complaints in perimenopausal women.

## Introduction

Menopause, marked by the loss of ovarian function and reduced sex hormones, leads to a wide range of physical and neurological symptoms. It is crucial to understand concomitant changes in the central nervous system to allow for the development of targeted therapies that improve postmenopausal women’s quality of life (Giannini et al., 2021). The menopause transition involves significant neuroendocrine changes driven by hormone fluctuations and estradiol withdrawal, which may disrupt central nervous system balance and contribute to mood changes and other menopausal symptoms through complex, interconnected signaling pathways (Fidecicchi et al., 2024). Menopausal and perimenopausal conditions affect millions of women worldwide, often causing a range of symptoms such as hot flashes, mood swings, sleep disturbances, and bone density loss (Delamater and Santoro, 2018; Santoro et al., 2021). In a review of 53 longitudinal studies involving more than 450,000 women, Andrews et al. (2024) found that menopausal symptoms are also important indicators for future health risks, including cardiovascular, psychiatric, and metabolic conditions.

Vasomotor symptoms affect most women during menopause and vary by individual factors, with multifactorial causes and a range of treatment options - hormonal and non-hormonal - requiring personalized, collaborative decision-making to balance benefits and risks (Atwell et al., 2024). Symptoms such as hot flashes may stem from a complex polygenic and molecular background, which can be influenced by diet-related bioactive compounds that modulate key signaling pathways, highlighting the potential for natural therapeutic approaches during menopause (Forma et al., 2024). A review of the literature (Swan and Alexander, 2024) concludes that despite challenges in evaluating dietary supplements for vasomotor symptoms due to the variability in products and limited clinical trials, evidence supports certain nonhormonal options like phytoestrogens, black cohosh, *Rheum Rhaponticum* (ERr 731®) and n-3 fatty acids, which may offer symptom relief with low risk when appropriately used. Rhubarb is one of the most ancient herbs in traditional Chinese medicine with diverse pharmacological properties (Xiang et al., 2020). Since the 1950s, an extract from the roots of rhapontic rhubarb (*Rheum rhaponticum* ERr 731®; trade name Phytoestrol® N) has been used in Germany for the treatment of follicle hormone disfunction in women with dysmenorrhea or climacteric complaints (Heger et al., 2023). ERr 731® is a standardized extract derived from the roots of *R. rhaponticum*, characterized by a defined composition of hydroxystilbene compounds. The principal constituents are the glycosides rhaponticin and desoxyrhaponticin and their corresponding aglycones rhapontigenin and desoxyrhapontigenin, all of which share a stilbene backbone structurally related to resveratrol. These compounds represent the pharmacologically relevant fraction of the extract and distinguish ERr 731® from anthraquinone-containing rhubarb preparations. This composition is consistent with its estrogen receptor–related activity profile, as stilbene derivatives are known to interact with estrogen signaling pathways in a subtype-selective manner (Vollmer et al., 2010). Overall, ERr 731® can be classified as a stilbene-rich phytopharmaceutical, with rhaponticin-derived constituents representing its key bioactive components that underpin its pharmacological effects (Heger et al., 2006).

A recent meta-analysis showed that ERr 731® significantly reduces menopausal symptoms (Dubey et al., 2024). While some endocrine disrupting chemicals like estrogen have been put on the so-called watch list related to the enforcement of the Drinking Water Directive of the European Union, rhapontic rhubarb can be considered safe for drinking water and may therefore be considered a future-oriented alternative to such constituents. Therefore, while estrogenic compounds may pose environmental and regulatory challenges, no such concerns exist for rhapontic rhubarb and its derivatives.

Hormone replacement therapy (HRT) is a common treatment for menopausal symptoms but carries potential hazards, including an increased risk of breast cancer and cardiovascular events (Madsen et al., 2023). Menopause is also linked to biological aging, and HRT is associated with biological rejuvenation in postmenopausal women (Deutsche Gesellschaft für Gynäkologische Endokrinologie und Fortpflanzungsmedizin, 2025; Liu and Li, 2024). Therefore, many women seek alternative, non-hormonal therapies to manage their symptoms effectively and safely (Pop et al., 2023; Zhang and Yang, 2025). To demonstrate the clinical relevance of ERr 731® in addressing common climacteric symptoms of menopause, namely, headaches/migraines, dizziness, paresthesia, and fluor vaginalis, a 12-week double-blind study and a 52-week multi-centric, prospective, open observational study were performed. The present paper describes the findings from this extended data collection.

## Methods

This study was a 12-week multi-centric, prospective, randomized, double-blinded (DB), phase III clinical trial on ERr 731® compared to placebo in patients with climacteric complaints in perimenopause. The trial was conducted using a three-stage group sequential design with adaptive sample size adjustments implemented at two interim analyses. The corresponding one-sided significance thresholds for the first, second, and final stages were α_1_ = 0.00026, α_2_ = 0.00710, and α_3_ = 0.02253, with critical values of 3.471, 2.454, and 2.004, and information rates of 0.333, 0.667, and 1.0, respectively.

**FIGURE 1:**
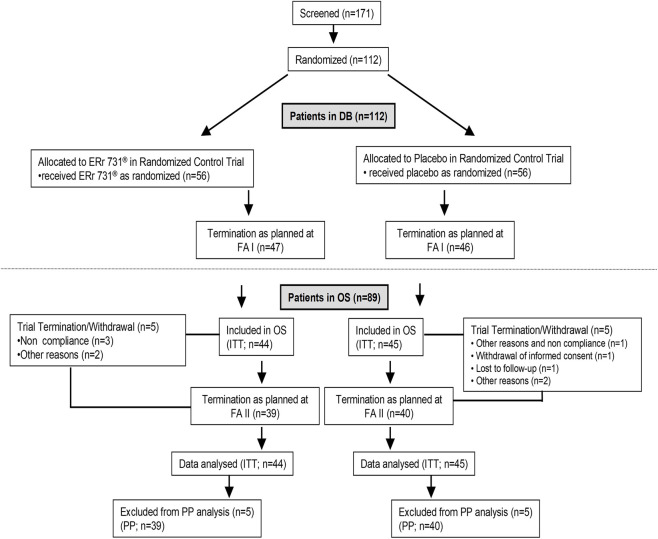
Study flow chart: 112 patients have been included in the RCT comparing the effect of ERr 731® intake to a placebo while 89 patients continued with the non-interventional OS where all patients received ERr 731®.

After the 12-week multi-centric trial, patients were offered to participate in a 52-week multi-centric, prospective open label observational study (OS) where those patients who were previously randomized to the ERr 731® group continued intake of the study product while those previously randomized to the placebo group started intake of ERr 731®. The study flow chart (Figure 1) provides an overview of the initial double-blind study and the OS for which the results are reported here. A total of 8 sites participated in this study, which was conducted in accordance with the ethical requirements of the Declaration of Helsinki, ICH GCP guideline, and the legal provisions of the Ukraine. Approval by the Ethics Committee, Kiev, Ukraine, and the State Pharmacological Committee of the Ministry of Health, Kiev, Ukraine, was obtained in December 2003 and the study was conducted between 2004 and 2007.

Detailed information on the study design and conduct as well as the inclusion and exclusion criteria can be found elsewhere (Kaszkin-Bettag et al., 2009). All patients included in this study were females aged 45–55 years in their perimenopause who displayed an MRS total score ≥18 points, were capable of providing written informed consent, were accessible by telephone, and were willing and able to comply with all procedures of the study and attend all scheduled contacts at the investigational sites.

During the 52-week OS, every 13 weeks observational contact with each study participant took place on days 91, 182, 273, and 364. Patients kept a patient diary in which they recorded their intake of the investigational medication and had to assess her general wellbeing every week by answering the question “How have you been feeling in general during the last week?” with one out of six given descriptions. The study was terminated with a final assessment. ERr 731® was administered through enteric coated tablets. The dose regimen was the same for all participating patients: 1 enteric coated tablet (ERr 731®) per day had to be taken orally at 8:00 o’clock in the morning, after breakfast. Each tablet (400 mg) contained 4 mg *R. rhaponticum* dry extract as the only active ingredient (drug:extract ratio 16–26:1, extraction solvent calciumoxide:water, 1:38 [m/m]).

The following climacteric complaints were evaluated in this study: headache/migraine, dizziness, paresthesia, fluor vaginalis, and general wellbeing. The listed symptoms were rated by the investigators at each examination time on a 5-point scale (0 = none, 1 = mild, 2 = moderate, 3 = severe, 4 = very severe). During all follow-up visits, safety parameters, and additionally, treatment compliance and termination of treatment according to the protocol were investigated. The investigator had to be the same for all assessments of a patient during the study.

Descriptive statistical methods were used to analyze all evaluated parameters. Explorative *p*-values were calculated for the comparison of ERr 731® between day 0 and day 84 of the RCT and between day 0 and day 364 of the OS using a two samples t-test.

## Results

In the initial randomized controlled trial (RCT), 171 women were screened for inclusion with 112 of them being enrolled. One site failed to enroll any patients, and participating women were therefore enrolled at 7 different investigational sites. Per protocol (PP) analysis was performed on 107 subjects while intention-to-treat (ITT) analysis of the results included 112 study participants. 89 patients agreed to participate in OS and were included and analyzed as treated (ITT population = 89; 44 patients from the previous ERr 731® group, 45 patients from the previous placebo group). A total of 89 out of 93 patients who participated in the initial RCT were subsequently included in the OS. Five of the 44 patients from the previous ERr 731® group and five of the 45 patients from the previous placebo group were excluded from the ITT population and analyzed per-protocol (PP population = 79).

### Safety

Overall, no serious adverse events (SAEs) or adverse events (AEs) with a causal relationship to the investigational medication were observed during this study.

### Headache/migraine

**FIGURE 2:**
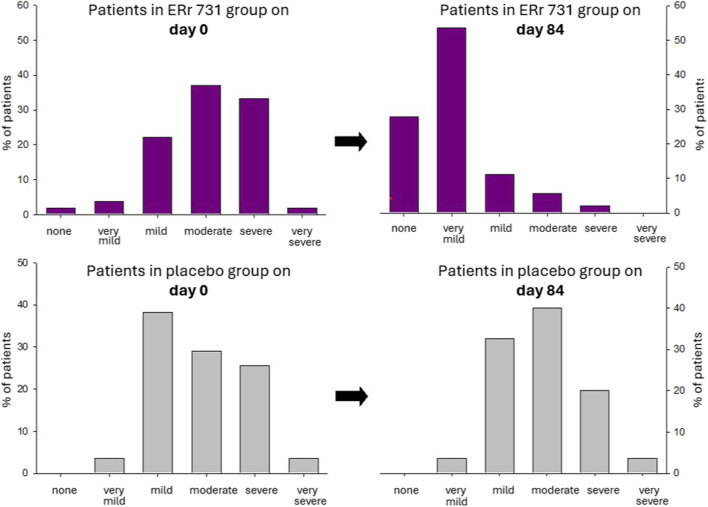
Assessment of headache/migraine during the RCT for patients in the intervention (ERr 731®) and the control group (placebo).

In the 12-week confirmation trial as well as in the subsequent 52-week observational study phase improvements in headache/migraine could be detected. At baseline (day 0), the severity of headache/migraine was comparable in both treatment groups (Table 1). From baseline (day 0) to the end of DB (day 84), the severity of headache/migraine decreased by −1.6 ± 0.9 (-2.0) points in the ERr 731® group and by −0.2 ± 0.8 [0,0] points in the placebo group resulting in a highly significant difference (*p* < 0.0001) in the severity as well as in the decrease of symptoms between the two treatment groups (Figure 2).

**TABLE 1:** Change in menopausal symptoms after 12 weeks of taking ERr 731^®^ or placebo. Shown are the scores for each symptom on day 0 and day 84 as mean values ± standard deviation and the difference between ERr 731^®^ and placebo on day 84 (mean values ± standard deviation [95% confidence interval]).

Symptoms	Change in the number of points from day 0 to day 84		ERr 731® minus placebo on day 84 [95% confidence interval]	
	ERr 731® (n = 44)	Placebo (n = 45)		
Headache/Migraine				
Day 0	2.5 ± 0.7	2.3 ± 0.9	​	​
Day 84	0.9 ± 0.6	2.1 ± 0.8	−1.22 ± 0.7***	[-1,540; -0.909]
Dizziness				
Day 0	2.0 ± 0.8	1.9 ± 0.8	​	​
Day 84	0.5 ± 0.7	1.7 ± 0.9	−1.26 ± 0.8***	[-1,587; -0.925]
Paresthesia				
Day 0	1.9 ± 1.0	1.8 ± 1.1	​	​
Day 84	0.6 ± 0.7	1.8 ± 1.1	−1.16 ± 0.9***	[-1,559, -0.770]
Vaginal discharge				
Day 0	1.6 ± 0.8	1.4 ± 0.7	​	​
Day 84	1.3 ± 0.8	1.3 ± 0.8	−0.05 ± 0.8n.s	[-0.294; 0.397]

*p < 0.05; **p < 0.001; ***p < 0,0001 (2 Sample t-test for the comparison of ERr 731® to Placebo), n.s., not significant.

At the end of the OS, there were remarks for 43/44 patients of the previous ERr 731® group and for 40/45 patients of the previous placebo group. Between day 0 of this OS and day 364 of this OS, the severity of headache/migraine decreased by −0.4 ± 0.6 (0.0) points in the previous ERr 731® group and by −1.4 ± 0.9 [-1.0] points in the previous placebo group (Table 2). By the end of the observational study phase, the difference in severity of headache/migraine between the two groups was no longer statistically significant (*p* = 0.1092). However, the reduction in symptoms from day 0 until day 364 of the OS was highly significant (*p* < 0.0001).

**TABLE 2:** Change in menopausal symptoms after 52 weeks of taking ERr 731^®^ or placebo. Shown are the scores for each symptom on day 84 and day 364 as mean values ± standard deviation and the difference between the previous ERr 731^®^ and placebo groups on day 364 (mean values ± standard deviation [95% confidence interval]).

Symptoms	Change in the number of points from day 84 to day 364		ERr 731® minus placebo on day 364 [95% confidence interval]	
	ERr 731® (n = 44)	Previous placebo (n = 45)		
Headache/Migraine				
Day 84	0.9 ± 0.6	2.1 ± 0.8	​	​
Day 364	0.5 ± 0.6	0.7 ± 0.5	−0.19 ± 0.6***	[-0.420; 0.043]
Dizziness				
Day 84	0.5 ± 0.7	1.7 ± 0.9	​	​
Day 364	0.2 ± 0.5	0.5 ± 0.5	−0.29 ± 0.5***	[-0.496;-0.074]
Paresthesia				
Day 84	0.6 ± 0.7	1.8 ± 1.1	​	​
Day 364	0.3 ± 0.6	0.6 ± 0.7	−0.24 ± 0.6***	[-0.496; 0.022]
Vaginal discharge				
Day 84	1.3 ± 0.8	1.3 ± 0.8	​	​
Day 364	1.3 ± 0.8	1.1 ± 0.7	0.18 ± 0.8^n.s^	[-0.139; 0.506]

*p < 0.05; **p < 0.001; ***p < 0.0001 (2 Sample t-test for the comparison of ERr 731® to placebo), n.s., not significant.

### Dizziness

At the beginning of the RCT on day 0, all patients in both treatment groups reported experiencing moderate dizziness or a sensation of dizziness (Table 1). After 12 weeks, the severity of dizziness in the ERr 731® group decreased by −1.5 ± 1.0 [-2.0] points and by −0.2 ± 0.5 [0,0] points in the Placebo group resulting in a highly significant difference in the severity as well as in the decrease of symptoms between the two treatment groups (*p* < 0.0001). In the ERr 731® group 27 patients had none and 14 mild dizziness, whereas in the placebo group had 24 moderate and 7 severe dizziness.

At the end of the RCT/beginning of the OS, 41/44 (93.2%) patients of the previous ERr 731® group indicated to have no or mild symptoms of dizziness compared to 14/45 (31.1%) patients of the previous placebo group. At the end of the OS, there were remarks for 43/44 patients of the previous ERr 731® group and for 40/45 patients of the previous placebo group regarding dizziness. The majority of patients of the previous ERr 731® group (41/43 patients) and all patients of the previous placebo group had no or mild symptoms.

Between day 0 of the OS and day 364 of this OS, the severity of dizziness reached 0.2 ± 0.5 [0.0] points in the ERr 731® group and 0.5 ± 0.5 [0.0] points in the placebo group. Thus, at the end of the observational study phase, the difference in severity of dizziness between the treatment groups was statistically significant (*p* = 0.0086) and the difference in decrease of symptoms from day 84 until day 364 was highly significant (*p* < 0.0001).

### Paresthesia

The results of the RCT show that after 12 weeks, the severity of paresthesia in the ERr 731® group decreased by −1.3 ± 1.0 [-1.0] points and remained constant in the Placebo group (−0.0 ± 0.7 [0,0]) resulting in a highly significant difference in severity as well as in the decrease of symptoms between the two treatment groups (*p* < 0.0001). In the ERr 731® group 21 patients had none and 20 mild paresthesia, whereas in the placebo group had 17 moderate and 14 severe paresthesia. At the end of the RCT/beginning of the OS, 41/44 (93.2%) patients of the ERr 731® group indicated to have no or mild symptoms of paresthesia compared to 14/45 (31.1%) patients of the placebo group.

At the end of the OS, remarks for 43/44 patients of the previous ERr 731® group and for 40/45 patients of the previous placebo group were available regarding symptoms of paresthesia. Most patients had no or mild symptoms (41/43 patients of the previous ERr 731® group and 37/40 patients of the previous placebo group). Between day 0 of the OS and day 364 of this OS, the severity of paresthesia reached 0.3 ± 0.6 [0,0] points in the previous ERr 731® group and 0.6 ± 0.7 [0.0] points in the previous placebo group. Therefore, at the end of the observational study phase (day 364), the difference in severity of paresthesia between the two treatment groups was no longer statistically significant (*p* = 0.0725). However, the reduction of symptoms from day 0 until day 364 of the OS was highly significant (*p* < 0.0001).

### Fluor vaginalis

On Day 0 of the trial, participants reported mild to moderate vaginal discharge (Table 1). Twenty-eight (28) out of 44 patients in the ERr 731® group had moderate and 8 mild vaginal discharge, whereas 20 out of the 45 patients in the placebo group had moderate and 18 mild vaginal discharge. After 12 weeks, the severity of vaginal discharge decreased slightly in patients in the ERr 731® group by −0.3 ± 0.6 [0,0] points while it remained nearly unchanged in the Placebo group (decrease: 0.1 ± 0.4 [0,0] points), both being 1.3 ± 0.8 points. The difference between the two treatment groups in the decrease of symptoms from Day 0 until Day 84 was not significant (*p* = 0.908). At the end of the RCT/beginning of the OS, 34/44 (77.3%) patients of the ERr 731® group had mild or moderate symptoms of fluor vaginalis compared to 35/45 (77.8%) patients of the placebo group. Ten (10) of the 44 (22.7%) patients in the ERr 731® group and 10/45 (22.2%) patients of the placebo group had no symptoms of fluor vaginalis.

At the end of the OS, there were remarks for 43/44 patients of the previous ERr 731® group and for 40/45 patients of the previous placebo group available. Most patients had mild or moderate symptoms (33/43 patients of the previous ERr 731® group and 32/43 patients of the previous placebo group). Between day 0 of the OS and day 364 of this OS, the severity of vaginal discharge was assessed at 1.3 ± 0.8 [1.0] points in the previous ERr 731® group and 1.1 ± 0.7 [1.0] points in the previous placebo group. Thus, at the end of the observational study phase, neither the difference in severity of vaginal discharge between the two treatment groups (*p* = 0.2621) nor the difference in the decrease of symptoms between day 0 and day 364 was statistically significant (*p* = 0.3227).

### General wellbeing

At baseline (day 0 of RCT), most patients (82/89 (92.1%) reported to be “up and down in spirits a lot” or to be “in low spirits mostly” (ERr 731®: 41/44 (93.2%), placebo: 41/45 (91.1%). At the end of the DB trial (Day 84), 25/44 (56.8%) patients in the ERr 731® group but only 5/45 (11.1%) patients in the placebo group reported to be “in good spirits mostly”. Most patients in the placebo group (39/45 (86.7%)) compared to 15/44 (34.1%) patients in the ERr 731® group reported to be “up and down in spirits a lot” or “in low spirits mostly”.

At the end of the RCT/beginning of the OS, 25/44 (56.8%) patients in the previous ERr 731® group but only 5/45 (11.1%) patients of the previous placebo group reported to be “in good spirits mostly”. Most patients of the previous placebo group (39/45 (86.7%)) but only some patients of the previous ERr 731® group (15/44 (34.1%)) reported to be “up and down in spirits a lot” or “in low spirits mostly”. At the end of this OS, most patients from both previous treatment groups reported to be “in very good spirits” or “in good spirits mostly” (previous ERr 731®: 40/44 (90.9%) patients; previous placebo: 43/45 (95.6%)). At the end of the study, most of the patients of the previous placebo group (39/45 (86.7%)) and 15/44 (34.1%) patients of the previous ERr 731® group reported an improvement in general wellbeing compared to the start of the OS. Most patients of the previous ERr 731® group (29/44 (65.9%) and 5/45 (11.1%) patients of the previous placebo group reported no further changes.

## Discussion

This study was aimed to assess the long-term efficacy of ERr 731® in perimenopausal women with climacteric symptoms. The study included a prospective RCT (Kaszkin-Bettag et al., 2009a; Kaszkin-Bettag et al., 2009b, Vollmer, et al., 2009) and an OS. The results presented here provide valuable insights into the efficacy and safety of ERr 731® as a non-hormonal alternative for managing climacteric symptoms, contributing to the growing body of evidence supporting its use in clinical practice. The data emphasizes the effectiveness of ERr 731® in reducing not only headaches and migraines, but also symptoms of paraesthesia and dizziness associated with menopause. A similar study set up was applied by Hasper et al. (2009) who evaluated the change in the Menopause Rating Scale II in an RCT followed by an OS. A decrease in symptoms could be found by this group of researchers if ERr 731® was taken by study participants. Similarly, at the end of the present study, the severity of headaches/migraines decreased in most of the participating patients, showing a highly significant improvement. Reproductive hormones, with estrogens in particular, may play a relevant role in the pathophysiology of migraine (Nappi et al., 2022). The estrogen withdrawal hypothesis is widely accepted as a trigger for menstrual migraine (Raffaelli et al., 2023a) and may be influenced by calcitonin gene-related peptide (CGRP) concentrations (Raffaelli et al., 2023b). A recent study by Verhagen et al. (2023) found that while the perimenstrual window increases the risk of migraine attacks without aura, it does not affect attacks with aura, suggesting that sex hormones differently influence migraine pain and aura mechanisms.

One potential cause for migraines is the release of inflammatory cytokines as e.g., interleukin (IL-)1β and IL-6, along with the production of reactive oxygen species due to oxidative stress, which induces inflammatory reactions. Cytokines stimulate the inducible NO-synthase (iNOS) in the dura mater, a pain sensitive intracranial tissue, leading to increased nitric oxide (NO) production (Reuter et al., 2002). Research has shown that estrogens can inhibit the expression of genes such as IL-6 and iNOS by binding to the NF-κB/RelA protein at specific promoter sites (Wen et al., 2004). In particular, the activation of ERβ appears to play a key role in suppressing the production of inflammatory mediators in microglia cells in certain areas of the brain (Baker et al., 2004). Since ERr 731® and its constituents are potent activators of ERβ, its activation might block the cytokine production, providing a crucial mechanism for migraine reduction. The successful application of rhapontic rhubarb in the treatment of headaches has also been documented by Struck (2013), who reported a case of a patient suffering from persistent complaints of dull pressure headaches, which could be relieved for only a few hours by the intake of Ibuprofen 600 mg. After the intake of rhapontic rhubarb for just 8 weeks, symptoms were reduced significantly with the patient being completely pain free on some days (Struck, 2013).

ERr 731® demonstrates pronounced functional selectivity for estrogen receptor-β (ERβ) over estrogen receptor-α (ERα) across multiple experimental systems. *In vitro* assays show that ERr 731® and its aglycones do not activate ERα in yeast, Ishikawa, or endometrial HEC-1B cells, whereas they significantly induce ERβ-dependent transcriptional activity at levels comparable to 17β-estradiol, confirming receptor-specific ERβ agonism (Wober et al., 2007). In subtype-specific analyses using U2OS osteosarcoma cells, ERr 731® exhibits strong ERβ activation but only weak ERα activity, which was also markedly lower compared to estradiol (Möller et al., 2007). This indicates a predominantly ERβ-mediated effect with limited ERα engagement depending on cellular context (Möller et al., 2007). These findings are supported by mechanistic evaluations demonstrating that, despite only modest differences in receptor binding affinity, ERr 731® produces stronger ERβ-dependent transcriptional responses, likely reflecting differential co-regulator recruitment (Vollmer et al., 2010). Collectively, the literature characterizes ERr 731® as a selectively acting, ERβ-preferential modulator of estrogen signaling with minimal ERα activation in estrogen-sensitive tissues, a profile that is consistent with its favorable safety observations, including no increased breast cancer risk in observational studies (Heger et al., 2025; Obi et al., 2009).

Beyond classical estrogen receptor signaling, ERr 731® may exert central effects via monoamine and neuroendocrine pathways. Hydroxystilbenes from *Rheum* species have been shown to inhibit monoamine oxidase-A (MAO-A) (Ryu et al., 1988; Wei et al., 2016), potentially increasing monoamine availability, which is relevant given elevated MAO-A activity and mood vulnerability during perimenopause (Newhouse et al., 2008; Rekkas et al., 2014). In parallel, ERr 731® preferentially activates ERβ (Vollmer et al., 2010), a receptor critically involved in stress regulation and synaptic plasticity (Cover et al., 2014; Handa et al., 2012), including hippocampal processes affected by stress (Kim and Diamond, 2002). Importantly, ERβ signaling is closely linked to oxytocinergic pathways, where it regulates oxytocin expression and contributes to anxiolytic effects (Acevedo-Rodriguez et al., 2015). Together, these findings suggest that ERr 731® may act not only via estrogen receptors but also through MAO-A inhibition and ERβ-linked oxytocin pathways, contributing to its effects on mood and stress-related menopausal symptoms.

In the present study, no significant reduction in vaginal discharge could be found in the present study, which may be associated with an ERr 731® induced enhancement of immune defense. Vaginal discharge caused by bacterial infections, Trichomonas vaginalis, or *Candida* albicans might be alleviated through the activation of immune cells. In ovariectomized ERβ-knockout mice, an increased release of TNF-α and a reduction in bacterial load due to heightened macrophage activity were observed, suggesting a potential role of ERβ in immune regulation (Lambert et al., 2004). Additionally, a study on 48 patients found that *R. rhaponticum* ointment was effective in the treatment of postmenopausal atrophy of labial and vaginal mucosa (Jaggi et al., 2016). Even women who had suffered from dryness, burning sensations, or a feeling of tightness for decades experienced significant relieve of those symptoms. The ointment with 0.1% *R. rhaponticum* (D2) (n = 37), 0.4% *R. rhaponticum* (n = 25) or a combined treatment with both concentrations was well tolerated by study participants.

Study participants who were treated with ERr 731® showed an improvement in their general wellbeing. A lack of interest or sensitivity towards both, private and professional environments was often accompanied by depressive moods in patients. As previously shown using the menopause-specific Quality of Life Questionnaires (MENQOL) (Heger et al., 2006), ERr 731® significantly improved these psychosocial issues. This is reflected in the improvement in the assessment of “insensibility towards family and/or profession”. Also, the self-assessment of patient’s health status using the EQ-VAS showed that just a 2-week intake of ERr 731® led to a significantly greater improvement in general health compared to placebo (Kaszkin-Bettag et al., 2009). The difference between ERr 731® and placebo continued to increase with longer treatment duration. In combination with results of other rating scales, namely, the Women’s Health Questionnaires and the general wellbeing (Psychological General Wellbeing Index (Kaszkin-Bettag et al., 2007)) patients confirmed the improvements in overall clinical impression observed by the investigators. These findings could be confirmed in the present study.

A relevant effect of ERr 731® intake in perimenopausal women seems to be the reduction of vasomotor and psychological symptoms, headache/migraine as well as potential inflammatory processes. The results have been confirmed in several clinical trials. Overall, rhapontic rhubarb has been used effectively in the treatment of menopausal symptoms and dysmenorrhea. The extract is characterized by its fast onset of effectiveness, also when treating mood swings, depression, and anxiety (Struck, 2010). The study results presented here show that the climacteric symptoms headache/migraine, dizziness, paresthesia, as well as general wellbeing could be improved with the intake of ERr 731®. Therefore, the 12-week RCT and the 52-week OS described here confirm the positive treatment results obtained with ERr 731® in perimenopausal women.

## Limitations

Some limitations should be considered when interpreting the study results presented here. Although the initial study phase was a randomized, double-blind, placebo-controlled trial, all participants received active treatment during the subsequent 52-week open-label observational phase. This eliminated the randomized comparison beyond week 12 and precludes long-term causal inference, as results from the observational phase may be influenced by non-randomized factors such as selection bias, expectation or placebo effects, and confounding by prior treatment exposure. In addition, symptom assessments relied partly on patient self-report and investigator-rated ordinal scales, which are susceptible to recall and reporting bias in an unblinded, long-term setting. While the study provides insight into sustained symptom trajectories under continued ERr 731® treatment, efficacy outcomes from the long-term phase should therefore be interpreted with caution. Finally, as the authors are owners or employees of Health Research Services GmbH, which funded the study, a potential sponsorship-related bias cannot be excluded.

## Conclusion

In this randomized controlled trial with extended observational follow-up, ERr 731® demonstrated significant short-term efficacy over placebo in reducing headache/migraine, dizziness, paresthesia, and improving general wellbeing in perimenopausal women with climacteric complaints. Improvements were sustained during 52 weeks of continued treatment, with symptom severity decreasing across most of the parameters assessed. These findings support ERr 731® as a well-tolerated, non-hormonal therapeutic option for the management of selected climacteric symptoms, particularly in women who seek alternatives to hormone replacement therapy. Given the observational nature of the long-term phase, future research should include long-duration randomized controlled trials to confirm sustained efficacy and safety.

## Data Availability

The raw data supporting the conclusions of this article will be made available by the authors, without undue reservation.
